# Selenium and RNA Virus Interactions: Potential Implications for SARS-CoV-2 Infection (COVID-19)

**DOI:** 10.3389/fnut.2020.00164

**Published:** 2020-09-04

**Authors:** Laurent Hiffler, Benjamin Rakotoambinina

**Affiliations:** ^1^Independent researcher, Lagny-sur-Marne, France; ^2^LRI Isotopic Medicine Lab, University of Antananarivo, Antananarivo, Madagascar

**Keywords:** selenium, COVID-19, SARS-CoV-2, thioredoxin reductase, RNA virus, elderly, obesity, oxidative stress

## Abstract

SARS-CoV-2 is an RNA virus responsible for the COVID-19 pandemic that already claimed more than 340,000 lives worldwide as of May 23, 2020, the majority of which are elderly. Selenium (Se), a natural trace element, has a key and complex role in the immune system. It is well-documented that Se deficiency is associated with higher susceptibility to RNA viral infections and more severe disease outcome. In this article, we firstly present evidence on how Se deficiency promotes mutations, replication and virulence of RNA viruses. Next, we review how Se might be beneficial via restoration of host antioxidant capacity, reduction of apoptosis and endothelial cell damages as well as platelet aggregation. It also appears that low Se status is a common finding in conditions considered at risk of severe COVID-19, especially in the elderly. Finally, we present a rationale for Se use at different stages of COVID-19. Se has been overlooked but may have a significant place in COVID-19 spectrum management, particularly in vulnerable elderly, and might represent a game changer in the global response to COVID-19.

## Introduction

SARS-CoV-2 is responsible for the COVID-19 pandemic that started in Wuhan, Hubei province, China and which already claimed more than 340,000 lives worldwide as of May 23, 2020 (1). It is a single-stranded RNA virus whose complex metabolism remains comparable to RNA viruses such as Influenza virus, some hemorrhagic fever viruses (such as Ebola virus or Hantavirus), Human Immunodeficiency Virus (HIV) and Coxsackievirus (2, 3). COVID-19 is a systemic disease and in addition to the more obvious respiratory symptoms, secondary symptoms that are now known to contribute to COVID-19 associated morbidity and mortality include heart problems like myocarditis (a primary feature of Coxsackievirus infection) (4) and systemic microthrombosis (a feature of hemorrhagic fever viruses) (5). Although few data are available thus far about the interaction between Se, an essential natural trace element, and SARS-CoV-2, RNA virus and Se interactions are well-documented (6–9). We present, firstly, a brief review of how Se deficiency promotes mutations, replication and virulence of different RNA viruses and secondly, the mechanisms by which Se could act in COVID-19 disease spectrum. Finally we discuss the potential links between host Se status and risk factors for severe COVID-19.

## Selenium Deficiency Promotes Mutations, Replication, and Virulence of RNA Viruses, and Selenium Has Clinical Benefit In RNA Viral Infections

### Coxsackievirus

Twenty years ago, Beck and Levander demonstrated that a usually mild Coxsackie virus could become highly virulent in Se deficient mice (6–8, 10). Keshan disease is a notable illustration. It is a myocarditis of viral origin (Coxsackievirus) occurring in a well-known Northeast/Southwest belt of China which is deficient in Se (11). Oxidative stress seems to be correlated with the degree of myocardial damage in patients with Keshan disease and selenoenzymes may also be involved in its pathogenesis (12). The prevalence of this viral disease, which at one aspect in time was responsible for thousands of deaths each year, has significantly reduced with the increased intake of Se (13).

### Hantavirus

Hantavirus (of the RNA Bunyaviridae family) can cause hemorrhagic fever with renal syndrome (HFRS) that is a group of clinically similar illnesses including Korean hemorrhagic fever, nephropathia epidemica and epidemic hemorrhagic fever (14).

In China, the incidence of HFRS is six times higher in Se-deficient areas (15). Viral replication was found to be higher in the event of Se deficiency and the intake of Se significantly inhibited the Hantavirus's replication at low viral titers (15). Remarkably, Hou (16) observed an 80% reduction in mortality in a cohort of 80 patients with Hantavirus epidemic hemorrhagic fever that were treated with a short 10 day intervention of high dose sodium selenite (16).

The combination of the last two cited studies are an important precedent which shows that, similar to Keshan disease/Coxsackievirus, a virus with increased incidence or severity in low Se regions responded clinically to a therapeutic intervention with a Se compound.

### Influenza Virus

During the H1N1 influenza pandemic, Moya et al. (17) showed that their group of patients with H1N1 pneumonia was more Se deficient than the control group (non-H1N1 but presenting influenza-like illness). In addition, patients with H1N1 pneumonia who had a blood Se level considered to be optimal for normal Glutathione Peroxidase (GPx) activity (18) recovered faster and had a better survival rate than the patients with H1N1 pneumonia but with lower Se levels (17).

Data in mice infected with the H1N1 virus and fed with either a Se deficient diet or supplemented with Se are striking: 75% mortality in Se deficient mice vs. 25% in Se supplemented mice (19). Similarly, Nelson et al. (20) showed that in Se deficient mice, increased viral mutations in the influenza virus A/Bangkok/1/79 (H3N2) genome were observed, resulting in a more virulent phenotype (20). Indeed, Se deficient mice and infected with H3N2 had more severe pulmonary histological damages than infected mice without Se deficiency.

### Human Immunodeficiency Virus Type 1 (HIV-1)

Unlike the above examples, in which the incidence, virulence or outcome of a viral infection was shown to correlate with Se status in specific geographic region, multiple reports over decades have shown that disease progression or mortality risk in HIV/AIDS is correlated with Se status in specific cohorts (21, 22). In addition, a therapeutic benefit of Se supplementation has been demonstrated in a number of clinical trials (23–26). Even in the case of HIV-1, one study was able to associate AIDS-related mortality in the US African-American population as of the year 1990 with Se status based on forage crop Se data (ranked as low, medium, and high) in different US States (27).

### Coronavirus SARS-CoV-2

COVID-19 is a multi-organ disease associated with a high morbidity and high rates of intensive care unit admissions. A recent epidemiological study by Zhang et al. (28) shows striking data comparing recovery rates from COVID-19 in 17 Chinese cities with population Se status (using hair Se data) for which they found a highly significant positive correlation (28): The lower the Se status in a population, the lower the recovery rate from COVID-19. Although this study does not prove causality, data are in the same line as other previously mentioned studies on RNA viruses and Se. However, one notable point in their results was that in the city of Enshi, which has one the highest Se intakes in the world, the recovery rate from COVID-19 was almost triple the average for the rest of the cities in Hubei Province, including Wuhan. This strongly suggests that the Se effect is not only of importance in Se deficient populations, but that having a higher than normal Se status may offer a protective benefit against the detrimental effects of the viral infection. This observation, like the Hou's study cited above for epidemic hemorrhagic fever (16), goes against the conventional wisdom that there is no benefit in Se supplementation above the minimum required dietary intake. These population based data have been recently confirmed in clinical practice in a study by Moghaddam et al. in Germany (29). They showed that serum Se levels were highly correlated with COVID-19 outcome in hospitalized patients. Very low serum Se status was present in 44.4% of their patients. Sixty five percent of the deceased had low Se vs. 39% in those who survived. The lowest serum Se were strongly associated with mortality.

## Mechanisms By Which Sodium Selenite Could Act In RNA Virus Infections Including SARS-CoV-2

### Restoration of the Host Cell Thioredoxin Reductase (TrxR) Biosynthesis

The TrxR is a major enzyme that contributes to DNA replication, defense against oxidative damage due to oxygen metabolism, and redox signaling (30). Taylor et al. (31) presented computational and *in vitro* evidence that HIV-1 and the Zaire strain of Ebola virus (EBOV) target cellular selenoprotein mRNAs by RNA:RNA antisense interactions for the purpose of hijacking the selenocysteine insertion sequence (“SECIS element”) of the cellular messenger RNA (mRNA) in order to express a viral selenoprotein (32). In both cases, the mRNAs of isoforms of thioredoxin reductase (TrxR) appear to be targeted. They also presented evidence in support of this mechanism via green fluorescent protein reporter gene assays, in which read-through of the 3'-UGA stop codons of the respective HIV-1 nef protein and EBOV nucleoprotein genes was evident (31). This mechanism would also be expected to lead to down regulation of the targeted TrxR protein, which could contribute to pathogenic effects of the virus, as it could result in increased oxidative stress (32).

Taylor and Ruzicka (33) later presented evidence of similar antisense targeting of several selenoprotein mRNAs, including selenoprotein P and TrxR1, by Zika virus mRNA, and proposed that the antisense knockdown of TrxR isoforms could be a general mechanism by which RNA viruses can increase the pool of ribonucleotides for viral RNA synthesis, by inhibiting the synthesis of DNA (33). Because of the essential role of TrxR in the thioredoxin system (30), which is a hydrogen donor for the reduction of ribose to 2'-deoxyribose, they proposed that this could be an effective strategy for RNA viruses. Because of the fact that TrxR is a selenoprotein in mammals, this may explain why various RNA viruses, including SARS-CoV-2, have shown a relationship to Se status, as reviewed above.

The antioxidant capacities of the host cell would certainly be affected by TrxR knockdown (9), which could be partially rectified by increasing Se intake to boost the availability of selenocysteine for selenoprotein synthesis. Sodium selenite is a rapidly bio-available form that can cross the blood-brain barrier more readily than other forms of Se. Pharmacological doses could help to rapidly restore the TrxR biosynthesis (34), thereby re-establishing cellular DNA synthesis and antioxidant capacities.

### Decrease in Viral-Induced Cell Apoptosis

Viral infection is associated with oxidative stress through the induction of enzymes that produce harmful reactive oxygen species (ROS) (9). There is an increase of ROS in a cell model infected with the influenza AH1N1 RNA virus (35). Sodium selenite decreases the production of ROS and the induced cell apoptosis in these infected cells (36).

Furthermore, Nuclear Factor *kappa*B (NF-*k*B) has anti-apoptotic or, on the contrary, apoptotic properties depending on the circumstances (37). RNA viruses can trigger activation of NF-*k*B and divert its function to their benefit (38, 39). Liao et al. (40) have shown that this NF-*k*B activation in cells infected with the nucleocapsid protein of SARS-CoV could be responsible, at least in part, for severe inflammatory pulmonary lesions in Severe Acute Respiratory Syndrome (40). In addition, NF-*k*B pathway activation is a key to various pro-inflammatory cytokines and chemokines production (41). Overproduction of these cytokines can lead to the life threatening “cytokine storm” (42, 43)

The inhibition of NF-*k*B induced by SARS-CoV in a mouse model is associated with greater survival (44). Se is well-established as an NF-*k*B inhibitor and may play a role in reducing viral-induced apoptosis (45–47) and might contribute to mitigate the cytokine storm linked with severe COVID-19 (48).

### Restoring the Host's Selenium Stock

Se is incorporated as selenocysteine into selenoproteins which exert immune and anti-inflammatory effects (49). At least 25 selenoproteins have been identified in humans, many of which are involved in antioxidant processes (9). Dietary Se supplementation is associated with increased selenoenzymes activity showing that baseline Se levels in some populations might be insufficient for optimal selenoenzymes activities (34, 50).

The use of Se in critically ill patients is a matter of controversy but this essentially applies to bacterial sepsis (51). Although COVID-19 may resemble sepsis, Se potential action in RNA related diseases is quite different (52). Indeed, RNA viruses, including the SARS coronaviruses, likely divert cellular Se for their own selenoproteins (9, 33, 53, 54). Intense viral replication would therefore induce a Se deficiency in the host cell (33, 55, 56). In this case, an exogenous supply of Se could restore the host “stock” that is essential for the biosynthesis of cellular selenoproteins, particularly those required for antioxidant defense.

### Selenium, in Particular as Selenite, Protects Endothelial Cells, and Acts on Blood Platelets Aggregation

The peculiar feature of COVID-19 is that it has been associated with thrombosis events such as large vessel clots, arterial adn deep vein thrombosis, pulmonary embolism, and microvascular thrombosis, probably due to endothelium dysfunction, platelet activation and inflammation (57, 58). It was recently demonstrated that SARS-CoV-2 induces endotheliitis as a result of both direct infection of endothelial cells in various organs and inflammatory response from the host (59, 60). Selenoenzymes such as Glutathione peroxidase and TrxR have an important role in the endothelial cell function (61). Oxidative damage can be reduced by selenite in human endothelial cells via a TrxR and GPx induction (62, 63).

COVID-19 is also associated with thrombocytopenia (64, 65) and an increasing incidence of strokes, even in younger patients, is a worrisome trend in COVID-19 pathology.

Regarding clot formation, Thromboxane A2 (TxA2) formation is a key factor in blood platelet activation and aggregation, resulting in blood coagulation/thrombosis formation and leading to recommendations as well as dilemmas of anticoagulant use in Intensive Care Units for COVID-19 patients (58, 66, 67). Sodium selenite has been shown to have an antiaggregating effect by decreasing TxA2 formation (68). In addition, higher serum Se levels are significantly associated with higher platelet count in healthy adults (69).

Hence, there is considerable potential for Sodium selenite to help reduce damage induced by endotheliitis and the systemic blood clotting that now appears to be a common and key feature of severe COVID-19 (70).

## Potential Relationship Between COVID-19 Outcome and Host Selenium Status

Se plays a key and complex role in the immune system especially in oxidative stress processes, via Thioredoxin, NADPH, and TrxR (49, 71). Moreover, it is now well-established that Se deficiency is associated with greater susceptibility to viral RNA infections and more severe outcome.

Besides other associated risk factors for severe COVID-19, Obesity (with Body Mass Index > 30 Kg/m^2^) is now clearly identified as major one (72, 73): the higher the BMI the higher the mortality risk. This important factor might contribute to the, to date, uncontrolled epidemic in the USA and Mexico where 42 % and 30 % of the population is obese, respectively (74, 75) and a relatively younger average age of the COVID-19 admitted patients compared to European cohorts (76). Most illnesses tied to worse outcomes in COVID-19 (77) are characterized by an underlying low-grade inflammation and oxidative stress such as obesity and metabolic syndrome (72, 78–81). Adipose tissue is a distinct endocrine organ and capable of synthesizing numerous cytokines such as IL-6, IL-8, and TNF-alpha (82). Obesity is characterized by increased macrophages infiltration in adipose tissue which is proven to be a SARS-Cov-2 reservoir (83). These macrophages can be activated locally by the virus leading to the deleterious production of reactive oxygen/nitrogen species if not counterbalanced by redox homeostasis (84, 85). Of note, Se attenuates pro-inflammatory gene expression in macrophages (86). miR-185-5p, a Se sensitive microRNA, was identified to regulate six of eight Glutathione Peroxidase and to control the feedback expression of selenoproteins in obese patients (87). Similarly, obesity is also associated with a reduction of Glutathione Peroxidase 3(GPx3) activity in adipose tissue (88). An recent animal study by Hauffe et al. demonstrated that sodium selenite supplementation in obese mice increased GPx3 activity and insulin receptors/sensitivity in adipose tissue (89). In addition, obesity is often accompanied by cardiovascular impairments such as endothelium dysfunction and arterial stiffness that could favor the endothelium infection by SARS-CoV-2 (90). Similarly, cardiac alterations and the prothrombotic propensity in obesity could increase the observed cardiovascular events in intensive care unit COVID-19 patients (58, 91, 92).

The fact that these pathologies exhibit oxidative stress and inflammation along with endothelium dysfunction and low Se level (93–97), and the relationships described above between Se and RNA viruses suggest a possible link between SARS-CoV-2 infection and Se.

In addition, compared with healthy adults, older subjects are consistently proven to be more vulnerable to COVID-19 with atypical clinical presentations (98, 99). They are at significantly increased risk of serious complications and mortality: average case fatality ratio of 4–5% above 60 years of age from international data, and probably up to 14.8% above 80 years (100, 101). Many factors could explain this such as blunted immune response, aging related frailty and multiple chronic co-morbidities along with a degree of malnutrition (102–105). Moreover, senescent fibroblasts are four times more prone to oxidative stress than young cells and consume more Se (106). Interestingly, the addition of Se in these cells, at high concentration, increases Glutathione Peroxidase activity and decreases reactive oxygen species (106). Low or borderline Se level in the elderly people is reported to influence longevity and mortality rate (107–110). In Sweden, 71% of elderly are Se deficient when admitted to Intensive Care Unit (111). Se supplementation has reduced significantly infections in institutionalized elderly people (110). Similarly, 4 year Se supplementation in Swedish elderly people reduced cardiovascular mortality risk by more than 40%, even 12 years after intervention (112).

## Discussion

In the first part, we reviewed the current evidence on how RNA virus can mutate, replicate and become more virulent in Se-deficient environment. There is now evidence that SARS-CoV-2 behaves similarly with the recent study from Zhang et al. (28) showing the link between COVID-19 recovery rate and the Se population status in 17 Chinese cities and Moghaddam et al. pointing at a strong correlation with low Se status and poor outcome in hospitalized patients (29) It is also important to note that SARS-Cov-2 Spike Protein S mutated (D614G) leading to an apparently more infectious strain now dominant in the Western regions as in the US (113, 114). Se presents various potential interests that can be applied to COVID-19 (115, 116). Evidence suggest that Se helps reducing oxidative stress by restoring selenoenzymes activities such as TrxR in particular (62). Its antioxidant effect would probably be most beneficial as prevention before one gets infected or at the very early stage before severe oxidative stress has arisen like in advanced severe COVID-19. In addition, Se plays a major role through its inhibitory effect on NFκB signaling, which acts as a central mediator of immune and inflammatory responses notably the pro-inflammatory molecules involved in life-threatening cytokine storm in COVID-19. Furthermore, Se protects endothelial cells function and may play a role in SARS-CoV-2 induced endotheliitis (61). Moreover, its antithrombotic properties are also recognized (68).

In many European countries populations, Se levels are below or borderline the level for optimal Glutathione Peroxidase and Selenoprotein P activity (117). It is estimated that up to a billion people are at risk of Se deficiency worldwide, and this is likely to increase with global warming (118). We have also shown that elderly, especially those with co-morbidities, are particularly prone to Se deficiency and oxidative stress (104, 108, 111) which may explain, in part, the worse outcome of COVID-19 in this subpopulation.

Sodium selenite (Na2SeO3) is readily available and is a compound already present in pharmacopeia in human diseases (in some countries as selenious acid, for addition to total parenteral nutrition solutions, as well as treatment of deficiency) and whose short-term toxicity is minimal, if not absent, and well-documented. It is rapidly incorporated and TrxR activity can increase in a few hours after supplementation (34, 111).

Pending the development of an effective vaccine, while faced with the deadly COVID-19 pandemic and especially the unprecedented pressure it exerts on the health care system, the potential use and effectiveness (or not) of sodium selenite must be urgently tested and documented in outpatient as well as inpatient settings.

Based on this review and summarized in Table 1, we propose a simple and readily feasible approach in line with the described clinical course of COVID-19 (100, 102, 119) consisting of:

- Prevention (Pr), in order to contribute coping with the disease: supplementing populations at risk of developing severe COVID-19 before they become infected. Priority target population would be represented by the elderly with co-morbidities and the obese. The issue of long term Se supplementation dosage has been controversial as its metabolism might involve a U-shape relation especially regarding glucose homeostasis (120). Therefore, long term supplementation needs special attention and it might be recommended to monitor glycemia after a few weeks. However, recent studies have been reassuring regarding dosages < 300 mcg notably for type 2 diabetes and prostate cancer (121–125). Long term 200 mcg Selenium supplementation in randomized controlled trials in HIV infection has been shown to be well-tolerated (24–26). In addition, 200 micrograms of Se daily has been given in the elderly for years with significant positive results lowering viral infection rates and cardiovascular mortality (110, 112). The American national institute of medicine recommends Se supplementations of <400 mcg/daily (126). Therefore, the approach of 200 mcg Se supplementation daily for 3 weeks followed by a maintenance dose of ≤ 200 mcg throughout the active circulation of SARS-Cov-2 seems safe and sound considering the high mortality risk related to COVID-19 in these vulnerable subgroups.
- Documentation of serum Se status whenever possible in hospitalized COVID-19 patients.
- Treatment (Th) of symptomatic patients: systematic addition of Se in symptomatic out patients at all ages and Sodium selenite upon hospital admission as soon as possible at the earliest stage before the deadly cytokine storm arises.

**Table 1:** Rationale for potential preventive (*Pr*) and/or therapeutic (*Th*) use of selenium (Se) in COVID-19.

**References**	**Main points**	**Potential Se application in COVID-19**
**RNA virus**		
(6, 8–11)	Se deficiency increases mutation, replication, and virulence of RNA viruses	As *Pr* in asymptomatic and *Th* in clinical settings for antiviral defense
(9, 30–32, 53, 54)	RNA viruses, including the SARS coronaviruses, likely divert cellular Selenium for their own selenoproteins and knockdown host TrxR/GPx (bioinformatic studies)	
(13)	Se supplementation decrease Keshan disease (linked to Coxsackie virus) prevalence	
(15, 16)	Se treatment reduced mortality Hantavirus infection (HFRS) in mice and humans	
(23–26)	Se supplementation HIV reduces disease progression and mortality	
(28, 29)	Se deficiency in population and patient-based studies associated with low recovery rate and mortality of COVID-19	
**Se general action**		
(86)	*In vitro* Se attenuates pro-inflammatory gene expression in macrophages	As *Pr* and *Th* of COVID-19 thrombosis and/or endothelitis and/or inflammatory complications
(57, 59)	*In vivo* Se supplementation increases platelet GPx activity and reduces aggregation through TXA2 inhibition and increased bleeding time	
(61, 62)	Se protects endothelial cells from oxidative insult	
(63)	*In vitro*, Se supplementation protects human endothelial cells from oxidative damage through TrxR and GPx induction	
(36, 45, 47)	Se mediates inhibition of the activation of the transcription factor NF-*k*B which regulates genes that encode inflammatory cytokines	Se (*Th*) might modulate NFκB activation in SARS-Cov-2 (cytokine storm)
(44)	in animal study, Se Inhibition of Nf-*k*B in SARS-Cov infected mice increased survival	
(35)	Se inhibits the production of ROS in H1N1 infected cell models	ROS effects, apoptosis oxidative stress (*Th*)
(36)	Se addition in cell media deficient in Se, counteracts ROS damages via GPx activity restoration	
**Obesity, elderly**		
(81)	Obesity related oxidative stress has deleterious impact on cardiovascular disease (Framingham study)	Obesity and elderly as independent risk factor of severe COVID-19
(80)	Obese hosts exhibit delayed and blunted antiviral responses to influenza virus infection	In both *Pr, Th* for complications: cardiovascular, endotheliitis, thrombosis, and to mitigate cytokine storm
(87)	In Bioinformatic study, Se deficiency alters miRNAs (miR-185-5p) that regulate selenoproteins expression (GPx) in oxidative stress and obesity	
(88, 89)	Obesity is tied to reduction of GPx3 activity in adipose tissue	In elderly, Se *Pr* as in asymptomatic and *Th* as soon as possible
	In animal study, Selenite treatment increases GPx3 activity in obese mice	
(96)	Serum Se level is significantly reduced among morbidly obese	
(106)	*ex vivo* study, Oxidative stress is highly increased in senescent fibroblasts that consume more Se. The addition of Se in these cells, increases GPx activity and decreases ROS	
(107, 110, 112)	In clinical study, In elderly subjects, the more Se deficiency the higher the mortality rate	
	In RCT, Se supplementation in elderly reduces viral events and cardiovascular mortality	

However, whichever stage of COVID-19, Sodium selenite might still have some benefits as an adjuvant therapy to tackle endotheliitis, coagulopathy and microthrombotic events and to restore some selenoenzymes activities reducing oxidative stress (61, 62, 68).

To counteract SARS-Cov-2 effects Se has to be given well above nutritional recommended daily allowance (RDA) i.e., 200 to 400 micrograms of Se element per day, which corresponds approximately to 600 to 1,200 micrograms of sodium selenite. Previous studies on Hantavirus infection and sepsis in ICU have demonstrated good tolerance of higher dosages of selenite for acute treatment (16, 51, 127). This would seem appropriate and safe for a very short period of two to three weeks in order to replenish selenium stock (29) in this impending life-threatening condition. In the hospital, the intravenous form is undoubtedly more suitable given a large number of digestive disorders in the context of COVID-19.

This mini review is about Se and its potential use in RNA virus with a focus on SARS-Cov-2. However, other factors have to be taken into account including at nutritional level. However, they do not fit in the scope of this review. We must cite for example the potential role of vitamin D, zinc, and poly unsaturated fatty acids (PUFA) that need proper studies and documentation in this context (128, 129).

## Conclusion

Se deficiency environments favor mutation, replication and virulence of RNA viruses. The fact that both, a link between population Se status in various Chinese cities and COVID-19 recovery rates has been described and, low serum Se status in German hospitalized COVID-19 patients has been associated with more severe outcome, are striking data showing that SARS-CoV-2 might be influenced by Se status, like other RNA viruses. Se induces antioxidant selenoenzymes activity and might rebalance the host response. Se as a NF-*k*B inhibitor plays a role in COVID-19 progression and acts as an immunomodulatory and anti-inflammatory micronutrient Se. Moreover, Se might reduce the effect of SARS-CoV-2 on vascular endothelial cells and aggregation of platelets. Oxidative state in COVID-19 is a common feature especially in elderly and obese patients both at risk of severe outcome. Here, by reducing oxidative stress, Se might have an important role to play. Sodium selenite is cheap and readily available. Pending the development of an effective vaccine, while faced with the deadly COVID-19 pandemic and especially the unprecedented pressure it exerts on the health care system, the potential use and effectiveness (or not) of sodium selenite must be urgently tested and documented in outpatient as well as inpatient settings. Similarly, as some countries enter the post-confinement era, implementing a preventive Se supplementation, especially in the elderly represent a sound, safe and feasible strategy. Se with its pleiotropic effects in COVID-19 disease sprectrum may become an important research topic in human health.

## Author Contributions

LH drafted the manuscript. LH and BR contributed equally on the final manuscript. All authors contributed to the article and approved the submitted version.

## Conflict of Interest

The authors declare that the research was conducted in the absence of any commercial or financial relationships that could be construed as a potential conflict of interest.

## Abbreviations

COVID-19: Coronavirus Disease 19 caused by SARS-CoV-2
DNA: Deoxyribonucleic acid
EBOV: Ebola virus
GPx: Glutathione Peroxidase
HIV: Human Immunodeficiency Virus
HFRS: Hemorrhagic fever with renal syndrome
mRNA: messenger Ribonucleic acid
NF-*k*B: Nuclear Factor *kappa*B
RNA: Ribonucleic acid
ROS: reactive oxygen species
SARS: severe acute respiratory syndrome
SARS-CoV-2: novel coronavirus 2019
Se: Selenium
TxA2: Thromboxane A2
TrxR: Thioredoxin reductase
3'-UGA: 3'- Uracil Guanine Adenine Stop codon.
